# Host species and site of collection shape the microbiota of Rift Valley fever vectors in Kenya

**DOI:** 10.1371/journal.pntd.0007361

**Published:** 2019-06-07

**Authors:** David P. Tchouassi, Ephantus J. Muturi, Samwel O. Arum, Chang-Hyun Kim, Christopher J. Fields, Baldwyn Torto

**Affiliations:** 1 International Centre of Insect Physiology and Ecology (*icipe*), Nairobi, Kenya; 2 Crop Bioprotection Research Unit, Agricultural Research Service, U.S. Department of Agriculture, Peoria, Illinois, United States of America; 3 Illinois Natural History Survey, University of Illinois at Urbana-Champaign, Champaign, Illinois, United States of America; 4 High Performance Computing in Biology (HPCBio), Roy J Carver Biotechnology Center, University of Illinois at Urbana-Champaign, Illinois, United States of America; University of Florida, UNITED STATES

## Abstract

The composition and structure of microbial communities associated with mosquitoes remain poorly understood despite their important role in host biology and potential to be harnessed as novel strategies for mosquito-borne disease control. We employed MiSeq sequencing of the 16S rRNA gene amplicons to characterize the bacterial flora of field-collected populations of *Aedes mcintoshi* and *Aedes ochraceus*, the primary vectors of Rift Valley fever (RVF) virus in Kenya. Proteobacteria (53.5%), Firmicutes (22.0%) and Actinobacteria (10.0%) were the most abundant bacterial phyla accounting for 85.5% of the total sequences. Non-metric multi-dimensional scaling plots based on Bray-Curtis dissimilarities revealed a clear grouping of the samples by mosquito species, indicating that the two mosquito species harbored distinct microbial communities. Microbial diversity, richness and composition was strongly influenced by the site of mosquito collection and overall, *Ae*. *ochraceus* had significantly higher microbial diversity and richness than *Ae*. *mcintoshi*. Our findings suggest that host species and site of collection are important determinants of bacterial community composition and diversity in RVF virus vectors and these differences likely contribute to the spatio-temporal transmission dynamics of RVF virus.

## Author summary

Knowledge of the microbial communities associated with disease vectors can be exploited for symbiotic control of vector-borne diseases. Here, we characterized and compared the bacterial communities of field-caught populations of *Aedes mcintoshi* and *Aedes ochraceus*, the primary vectors of Rift Valley fever (RVF) virus in Kenya. We show that the two mosquito species harbor distinct microbial communities whose diversity and richness are heavily influenced by the site of collection. Because some bacterial species are known to influence vector susceptibility to pathogens, differences in bacterial communities between the two mosquito species is likely one of the primary factors accounting for the spatial and temporal variation in transmission dynamics of RVF virus.

## Introduction

Rift Valley fever (RVF) is a mosquito-borne zoonotic disease caused by RVF virus (Bunyaviridae: *Phlebovirus*). Over the last several decades, the virus has caused periodic epizootics and epidemics in Africa, which have been associated with severe economic and nutritional impacts [1]. Due to its devastating effects and significant potential for international spread, RVFV is listed as a select agent with bioterrorism potential [2]. Efforts to prevent RVF are mainly devoted towards development of vaccines. Currently, there are no licensed RVF vaccines for use in humans [3] and those that are commercially available for use in livestock face many challenges that affect their effective utilization. These challenges include safety concerns, induction of abortion, fetal malformation in pregnant ewes, stillbirths, need for annual revaccination or application of multiple doses and the inability to differentiate naturally infected animals from vaccinated animals [3–5]. Moreover, the highly susceptible RVF hosts in greater need of vaccination such as sheep and goats have high population turn-over rates, limiting the maintenance of herd immunity especially in pastoralist areas [6]. The lack of safe and effective vaccines for medical and veterinary use underscores the urgent need for alternative strategies for RVF control particularly those targeting the vectors.

Mosquitoes serve as hosts for diverse microscopic life-forms including viruses, bacteria, fungi, and protozoa that are collectively referred to as microbiota. Bacteria are the best-studied component of mosquito microbiota, with some members known to inhibit transmission of mosquito-borne pathogens and to contribute essential functions in mosquito survival, development, and reproduction [7–13]. In addition, certain bacterial symbionts can be genetically transformed to secrete anti-pathogen molecules within the vector [14,15]. These findings have stimulated interest in the development of a novel vector-borne disease control strategy based on the application of microbial symbionts to reduce vector competence and suppress vector populations [16].

Although significant progress has been made towards the application of microbes such as *Wolbachia* for mosquito-borne disease control, we still lack the basic understanding of the natural microbial communities associated with diverse mosquito species of medical and veterinary significance. For example, while a great wealth of knowledge is available on microbial communities of mosquito vectors of malaria, dengue, Zika, Yellow fever, and West Nile virus [17–20], the microbiota of RVFV vectors remain poorly understood. The lack of this knowledge has limited our ability to understand the influence of mosquito microbiota on RVF transmission and their potential application in RVF disease prevention and control.

The objective of this study was to characterize the microbial communities of *Aedes mcintoshi* and *Aedes ochraceus*, the primary vectors of RVFV in Kenya [21,22]. *Aedes mcintoshi* and *Ae*. *ochraceus* are flood water mosquito species belonging to the subgenus *Neomelaniconion* and *Aedimorphus*, respectively. Large populations of the two mosquito species occur in major hot spots for RVF in Kenya [23] and RVFV has been isolated in pools of both mosquito species during outbreak periods [24]. Inter-epidemic maintenance of RVFV through transovarial transmission has also been documented in *Ae*. *mcintoshi* [25]. As primary vectors, these mosquito species have been reported to exhibit distinct genetic population structure and demographic patterns in relation to variable occurrence and outbreak patterns of RVF in different ecologies of Kenya [22]. To accomplish our objectives, mosquitoes were collected from four study sites and their microbial composition characterized through MiSeq sequencing of the 16S rRNA gene. Our results reveal that the two mosquito species have distinct microbial communities and that *Ae*. *ochraceus* has significantly higher microbial diversity and richness than *Ae*. *mcintoshi*. These results provide critical insights into the composition and structure of microbial communities of important disease vectors and may guide the identification of bacterial species that could be harnessed for symbiotic control of RVF virus.

## Materials and methods

### Study sites and mosquito collection

**Fig 1:**
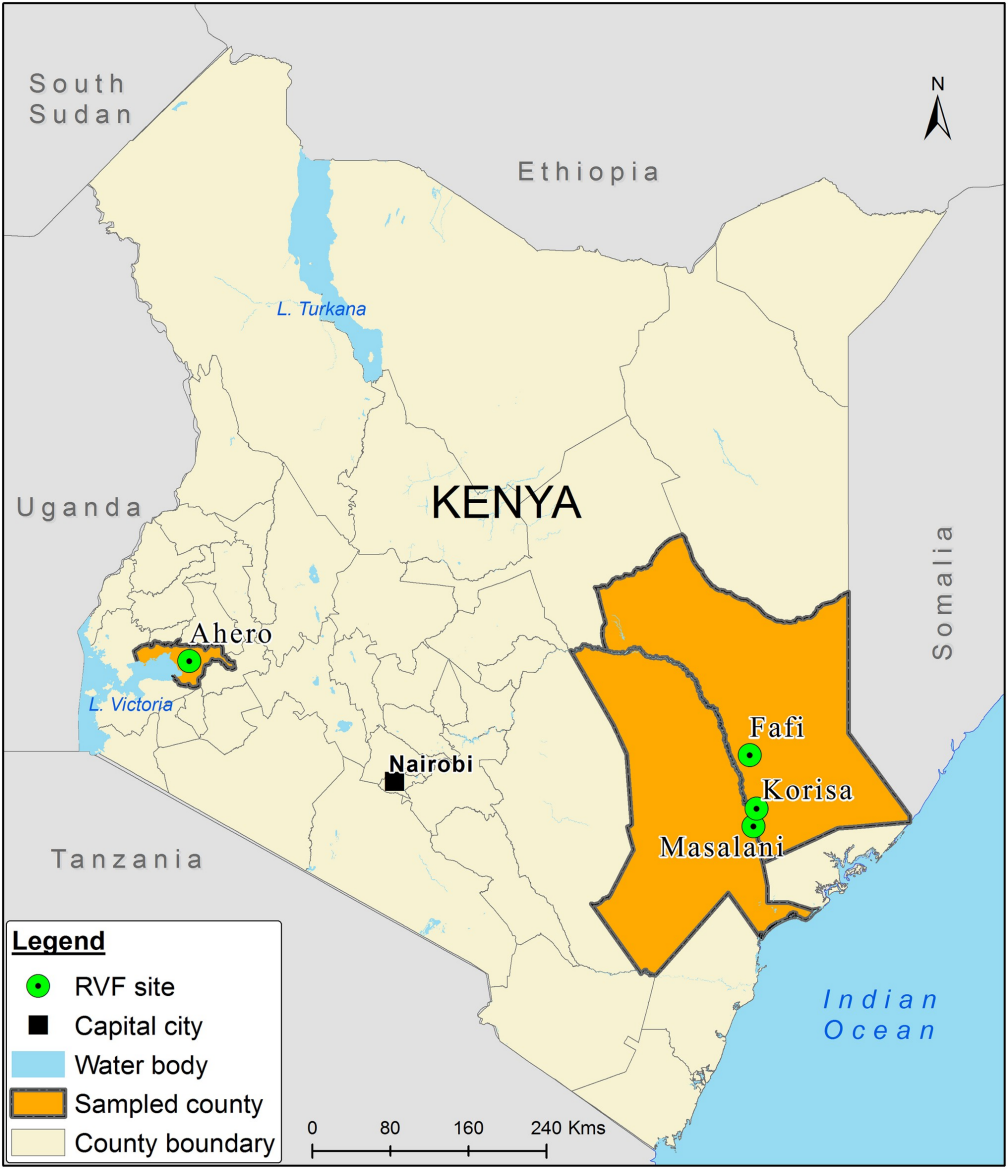
Map of Kenya showing mosquito sampling sites. The map was designed using ArcMap 10.2.2. with the ocean and lakes base layer derived from Natural Earth (http://www.naturalearthdata.com/, a free GIS data source). The sample points were collected using a GPS gadget (garmin etrex 20, https://buy.garmin.com/en-US/US/p/518046), and the county boundaries for Kenya derived from AfricaOpendata (https://africaopendata.org/dataset/kenya-counties-shapefile, license Creative Commons).

Adult females *Ae*. *mcintoshi* and *Ae*. *ochraceus* were sampled using CDC miniature light traps (John W. Hock Company, Model 512) baited with dry ice. Collections were made during the rainy season between November 2015 and June 2016 from four sites (Korisa, Masalani and Fafi in northeastern Kenya and Ahero in western Kenya) where the two mosquito species are known to occur [22,26] (Fig 1). The sites were selected as part of an on-going project monitoring the inter-epidemic circulation of RVF in these communities. Collections were conducted for two consecutive nights per site with traps operated between 1800 hours and 0600 hours. Trapped mosquitoes were anesthetized for 2 min using triethylamine before sorting and transporting in liquid nitrogen to the laboratory of the International Centre of Insect Physiology and Ecology (*icipe*), Duduville Campus, for storage at -80°C until further processing. Identification of *Ae*. *mcintoshi* and *Ae*. *ochraceus* was established with the aid of published taxonomic keys [27,28]. The number of samples processed per site are shown in Table 1.

**Table 1:** Information on sampling sites for the two mosquito species collected in Kenya.

				*Ae*. *mcintoshi* (n)		*Ae*. *ochraceus* (n)	
Sites	Region	Latitude	Longitude	Initial	Final	Initial	Final
Masalani	Northeastern	-1.697419	40.122873	19	18	20	20
Korisa	Northeastern	-1.534903	40.151456	20	18	5	5
Fafi	Northeastern	S01°02.460	E40°05.158	11	11	52	45
Ahero	Western	S00.174	E034.920	20	20	6	6

n, number of mosquito samples analyzed for each study site. Initial, no. of samples processed; final, no. of samples retained after removing sequences less than 900 reads

### Mosquito processing, DNA extraction 16S rRNA library preparation

Adult females that were not visibly blood-engorged were processed for microbial analysis. Prior to genomic DNA extraction, adult female mosquitoes were surface sterilized by rinsing them in 2% bleach solution for 1 min, followed by 70% ethanol for 5 min and then three rinses in sterile phosphate-buffered saline solution each lasting 10 sec. Genomic DNA was extracted from individual mosquitoes using the Qiagen DNeasy Blood and Tissue Kit (Qiagen, GmbH Hilden, Germany) following the manufacturer’s recommendations and stored at -20°C until further processing. In total, 153 mosquito samples from two mosquito species, *Ae*. *mcintoshi* (n = 70) and *Ae*. *ochraceus* (n = 83) were processed (Table 1). A similar sample size has been used in previous studies on mosquito microbiota [18,29].

All DNA samples were shipped to the W.M Keck Center for Comparative and Functional Genomics at the University of Illinois for sequencing using Illumina MiSeq Bulk V3 platform. The PCR amplification and sequencing procedures targeting the V3-V5 hypervariable region of bacterial 16S rRNA gene were performed as described in our previous reports [19,20,30]. In brief, all DNA samples were measured on a Qubit (Life Technologies) using High Sensitivity DNA kit and diluted to 2 ng/μl. PCR master mix was prepared using the Roche High Fidelity Start Kit and 20x Access Array loading reagent and aliquoted into 48 well PCR plates along with 1 μl DNA sample and 1 μl Fluidigm Illumina linkers (V3-V5-F357: ACACTGACGACATGGTTCTACA and V3-V5-R926:TACGGTAGCAGAGACTTG-GTCT) with unique barcode. In a separate plate, 20x solutions of the following primer pairs: forward 5`-CCTACGGGAGGCAGCAG-3`and reverse 5`-CCGTCAATTCMTTTRAGT-3`were prepared by adding 2 μl of forward and reverse primer, 5 μl of 20x Access Array Loading Reagent and water to a final volume of 100 μl.

A 4 μl aliquot of sample was loaded in the sample inlets and 4 μl of primer (20x) loaded in primer inlets of a previously primed Fluidigm 48.48 Access Array IFC, and samples on the array were then amplified on the Fluidigm Biomark HD PCR machine using the following Access Array cycling program without imaging: 50°C for 2 min (1 cycle), 70°C for 20 min (1 cycle), 95°C for 10 min (1 cycle), followed by 10 cycles at 95°C for 15 sec, 60°C for 30 sec, and 72°C for 1 min, 2 cycles at 95°C for 15 sec, 80°C for 30 sec, 60°C for 30 seconds, and 72°C for 1 min, 8 cycles at 95°C for 15 sec, 60°C for 30 sec, and 72° for 1 min, 2 cycles at 95°C for 15 sec, 80°C for 30 sec, 60°C for 30 sec, and 72°C for 1 min, 8 cycles at 95°C for 15 sec, 60°C for 30 sec, and 72°C for 1 min, and 5 cycles at 95°C for 15 sec, 80°C for 30 sec, 60°C for 30 sec, and 72°C for 1 min. The PCR product was transferred to a new 96 well plate, quantified on a Qubit fluorimeter (Thermo-Fisher) and stored at -20°C. All samples were run on a Fragment Analyzer (Advanced Analytics, Ames, IA) and amplicon regions and expected sizes confirmed. Samples were then pooled in equal amounts according to product concentration. The pooled products were size selected on a 2% agarose E-gel (Life Technologies) and extracted from the isolated gel slice with QIAquick gel extraction kit (QIAGEN). Cleaned size selected products were run on an Agilent Bioanalyzer to confirm appropriate profile and determination of average size. The final library pool was spiked with 10% non-indexed PhiX control library (Illumina) and sequenced using Illumina MiSeq V3 Bulk system. The libraries were sequenced from both ends of the molecules to a total read length of 300nt from each end. Cluster density was 964k/mm^2^ with 85.9% of clusters passing filter.

### OTU picking and taxonomy assignment

De-multiplexed FASTQ-formatted files obtained from the sequencing facility were processed using IM-TORNADO 2.0.3.2 platform which is specifically designed to process non-overlapping reads [31]. A detailed description of how raw reads were processed prior to OTUs assignment is provided in our previous report [20]. In brief, forward and reverse primers were removed prior to quality trimming, discarding reads with less than 150 base pairs. R1 and R2 reads were joined by the IM-TORNADO workflow. Sequences were clustered at 97% sequence similarity to generate operational taxonomic units (OTUs), and then representative sequences were classified taxonomically using the Ribosomal Database Project (RDP) version 10 as the reference set and mothur v 1.28 [32], with a threshold of 60% bootstrap confidence [31,33,34].

### Statistical analysis

All data were analyzed using R version 3.3.2, and PAST 3.20 statistical packages. There were marked variations in the number of bacterial sequences between mosquito samples (Mean ± SE = 27,796.01 ± 2,229.73 per mosquito, minimum = 5, maximum = 164,228). Only samples with at least 900 sequences (n = 143) were used for downstream analysis in R. Rarefaction curves for entire dataset were generated using “*phyloseq*” [35]. For additional analysis, OTUs accounting for less than 0.005% of the total sequences were discarded. Venn diagrams to visualize the OTUs that were shared between mosquito species and study sites were created using “*limma*” [36]. The difference in *Ae*. *mcintoshi* and *Ae*. *ochraceus* OTUs that were shared across study sites were compared using Fisher’s exact test implemented in the package *RVAideMemoire* [37]. Alpha diversity metrics for each sample including Shannon diversity index, observed OTUs (richness) and Chao1 were computed using *vegan* package after rarefying all samples to an even depth of 900 sequences [38]. We compared the relative OTU abundances across sites for each species using chi square good-ness-of-fit test implemented in the package *RVAideMemoire* [37]. Multiple pairwise comparisons across sites was performed using adjusted *P* after *false discovery rate* (*fdr*) correction [39]. Non-parametric Scheirer-Ray-Hare test was used to test for statistical significance and Wilcoxon rank sum test with Bonferroni correction for multiple comparisons was used to establish treatments that were significantly different. For beta diversity, we used unrarefied data as suggested by [35]. To minimize sampling bias, we transformed the raw dataset into proportion representing relative contribution of each OTU. This simple normalization provides a simple representation of the count data as a relative abundance measure [40]. Non-metric multidimensional scaling (NMDS) with Bray-Curtis dissimilarity metric was then performed in R package “*phyloseq*” [35] to visualize the effect of site and mosquito species on bacterial communities [41]. Results of NMDS were confirmed using analysis of similarities test (ANOSIM) with 9,999 permutations using PAST. Indicator species analysis was conducted using the R package ‘*labdsv*’ [42] to identify the bacterial OTUs that characterized each mosquito species based on fidelity and specificity [43]. Only OTUs with an indicator value ≥ 60% were considered significant.

### Ethics statement

Consent was sought from community elders or chiefs to set up traps away from homesteads on community land.

## Results

### Bacterial diversity and richness in *Ae*. *mcintoshi* and *Ae*. *ochraceus*

**Fig 2:**
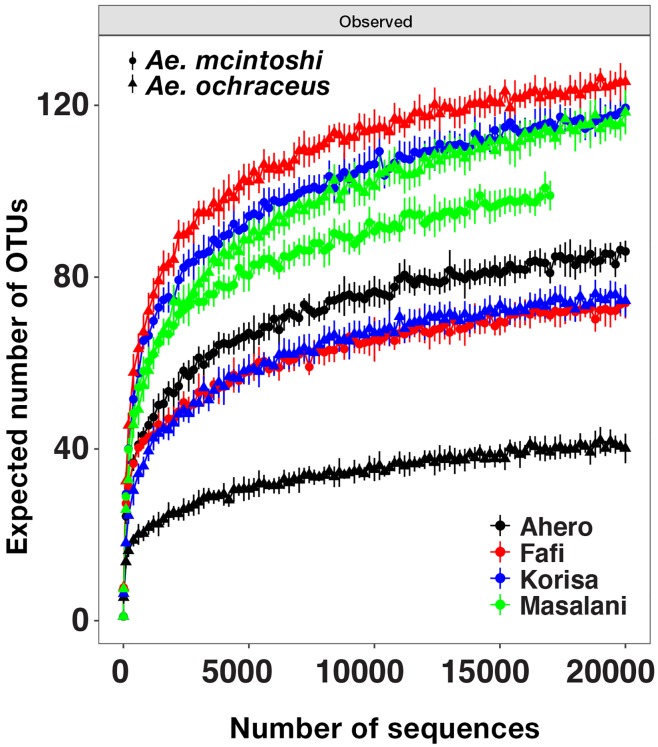
Rarefaction curves showing expected number of bacterial OTUs for the two mosquito species.

To survey the microbial communities of field-collected populations of *Ae*. *mcintoshi* and *Ae*. *ochraceus*, the V3-V5 hypervariable region of bacterial 16S rRNA was PCR amplified and sequenced using Illumina MiSeq platform. Rarefaction curves generated using all mosquito samples (n = 153) demonstrated that bacterial OTU richness for both mosquito species varied markedly across study sites (Fig 2). OTU richness was highest in *Ae*. *ochraceus* from Fafi and lowest in *Ae*. *ochraceus* from Ahero. Rarefaction curves indicated that sequencing coverage of the bacterial communities was adequate for some but not all samples.

**Fig 3:**
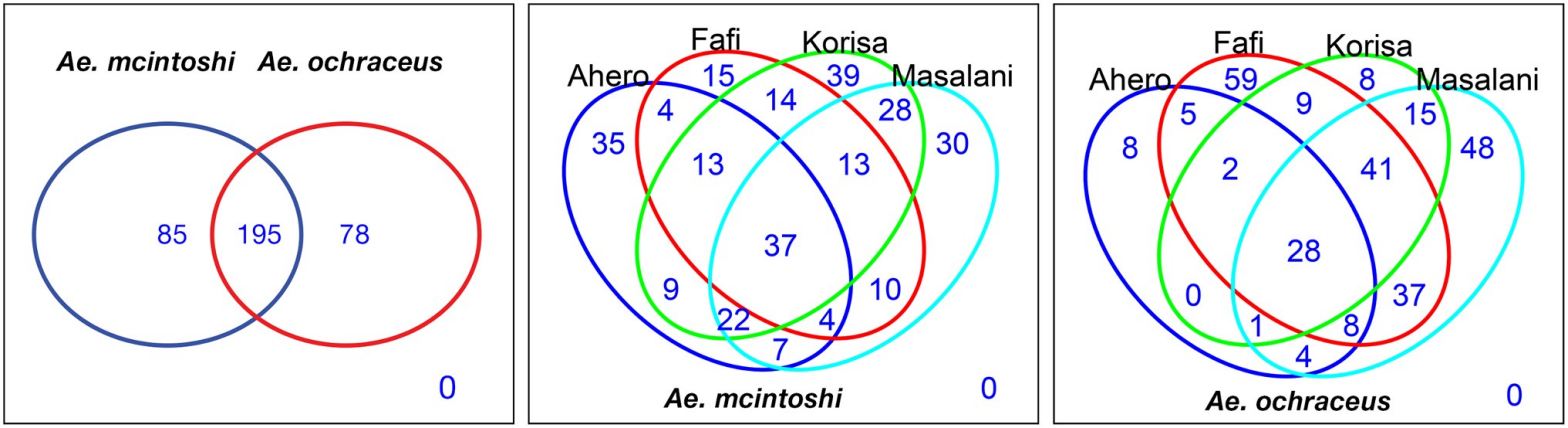
Number of shared and unique OTUs between *Aedes mcintoshi* and *Aedes ochraceus* and for each species between study sites.

Of the 358 OTUs that were detected in the two mosquito species, 85 and 78 OTUs were unique to *Ae*. *mcintoshi* and *Ae*. *ochraceus*, respectively and 195 were shared between the two mosquito species (Fig 3). For *Ae*. *mcintoshi*, 35, 15, 39, and 30 OTUs were unique to Ahero, Fafi, Korisa and Masalani, respectively, with only 37 (13%) common across the sites. For *Ae*. *ochraceus*, 8, 59, 8, and 48 OTUs were unique to Ahero, Fafi, Korisa and Masalani, respectively. There was a significant difference in unique OTUs between *Ae*. *mcintoshi* and *Ae*. *ochraceus* across the study sites (Fisher’s exact test, *P* <0.0001). Significant differences were observed between Ahero and Korisa (*P* <0.0001), Ahero and Masalani (*P* <0.0001), Fafi and Korisa (*P* <0.0001) and Fafi and Masalani (*P* <0.0001). Only 10% of the OTUs (28 out of 273) were shared across the study sites in *Ae*. *ochraceus*. Overall, the proportion of shared OTUs among sites for both species were comparable (*Ae*. *mcintoshi*: 13%, 37/280; *Ae*. *ochraceus*: 10%; 28/273; Fisher’s exact test, *P* = 0.5082). The proportion of OTUs unique to specific study sites was also comparable between the two mosquito species (*Ae*. *mcintoshi* vs *Ae*. *ochraceus*: 119/280 vs 123/273; Fisher’s exact test, *P* = 0.5498). For *Ae*. *mcintoshi*, the number of OTUs exclusively shared between any pair of sites was lowest between Ahero and Fafi (4) and highest between Korisa and Masalani (28). For *Ae*. *ochraceus*, the lowest number of OTUs that were shared between any pair of sites was lowest in Ahero and Korisa (0) and highest between Fafi and Masalani (37).

**Fig 4:**
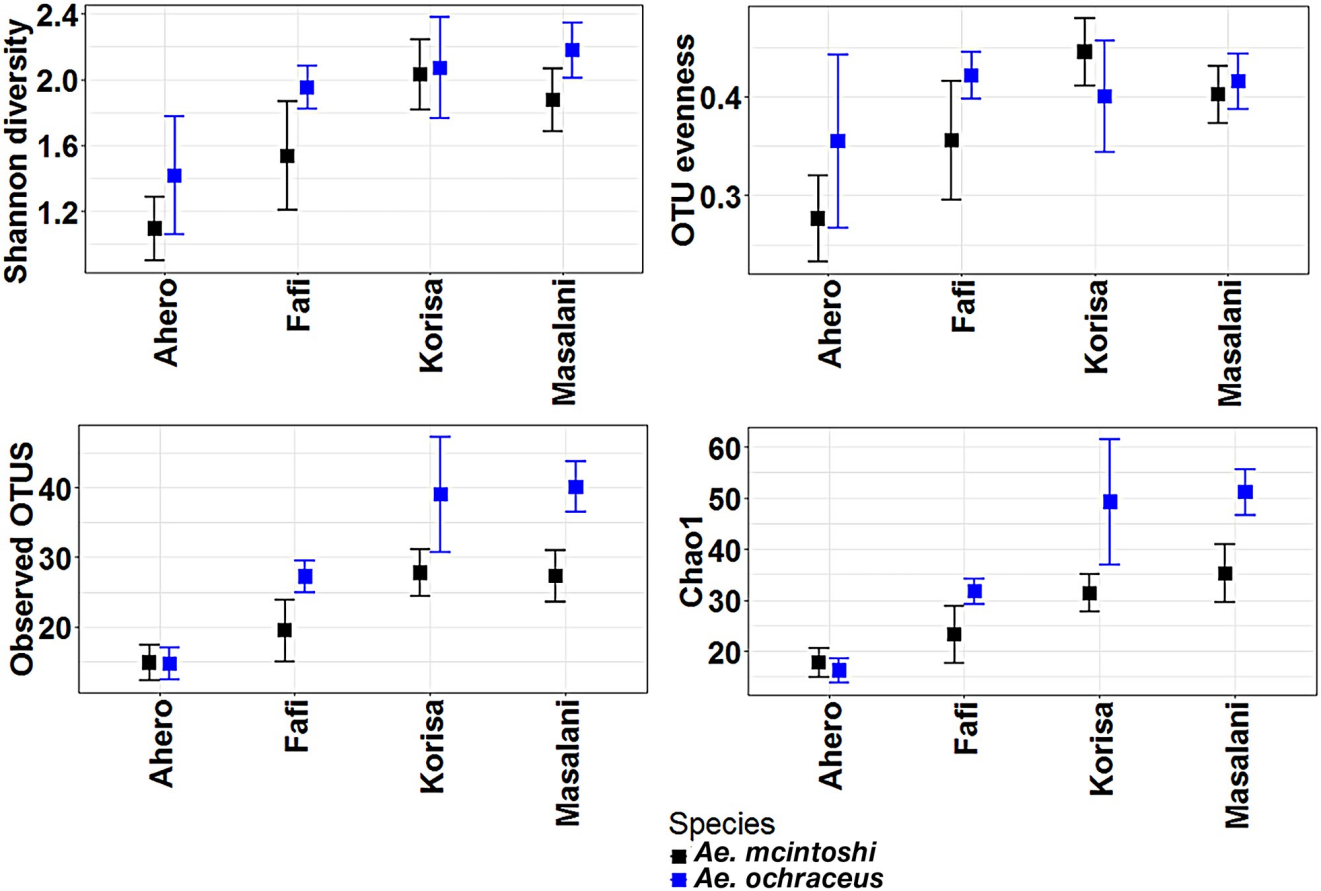
Biodiversity estimators of *Aedes mcintoshi* and *Aedes ochraceus* microbiota (mean ± standard error).

Shannon diversity index, observed OTU and Chao1 but not evenness were significantly influenced by host species and site of collection but not their interaction (Table 2). *Ae*. *ochraceus* had significantly higher microbial richness and diversity compared to *Ae*. *mcintoshi* (Fig 4). For the site effect, Shannon diversity index, observed OTUs, and Chao1 values were significantly lower in Ahero compared to the other sites. Chao1 values were also significantly lower in Fafi compared to Masalani.

**Table 2:** Scheirer-Ray-Hare test results for the effect of mosquito species and site of collection on alpha diversity indices.

		df	Sum Sq	H	*P* value
**Shannon**	Species	1	9512	5.54	0.019
	Site	3	21480	12.52	0.006
	Species x Site	3	499	0.29	0.961
	Error	135	212181		
**Evenness**	Species	1	3619	2.11	0.146
	Site	3	11392	6.64	0.084
	Species x Site	3	3162	1.84	0.606
	Error	135	22598		
**Observed**	Species	1	13899	8.11	0.004
	Site	3	40741	23.76	<0.0001
	Species x Site	3	2055	1.20	0.753
	Error	135	186768		
**Chao1**	Species	1	15089	8.79	0.003
	Site	3	42097	24.54	<0.0001
	Species x Site	3	3711	2.16	0.539
	Error	135	182738		

### Taxonomic classification of bacteria in *Aedes mcintoshi* and *Aedes ochraceus*

**Fig 5:**
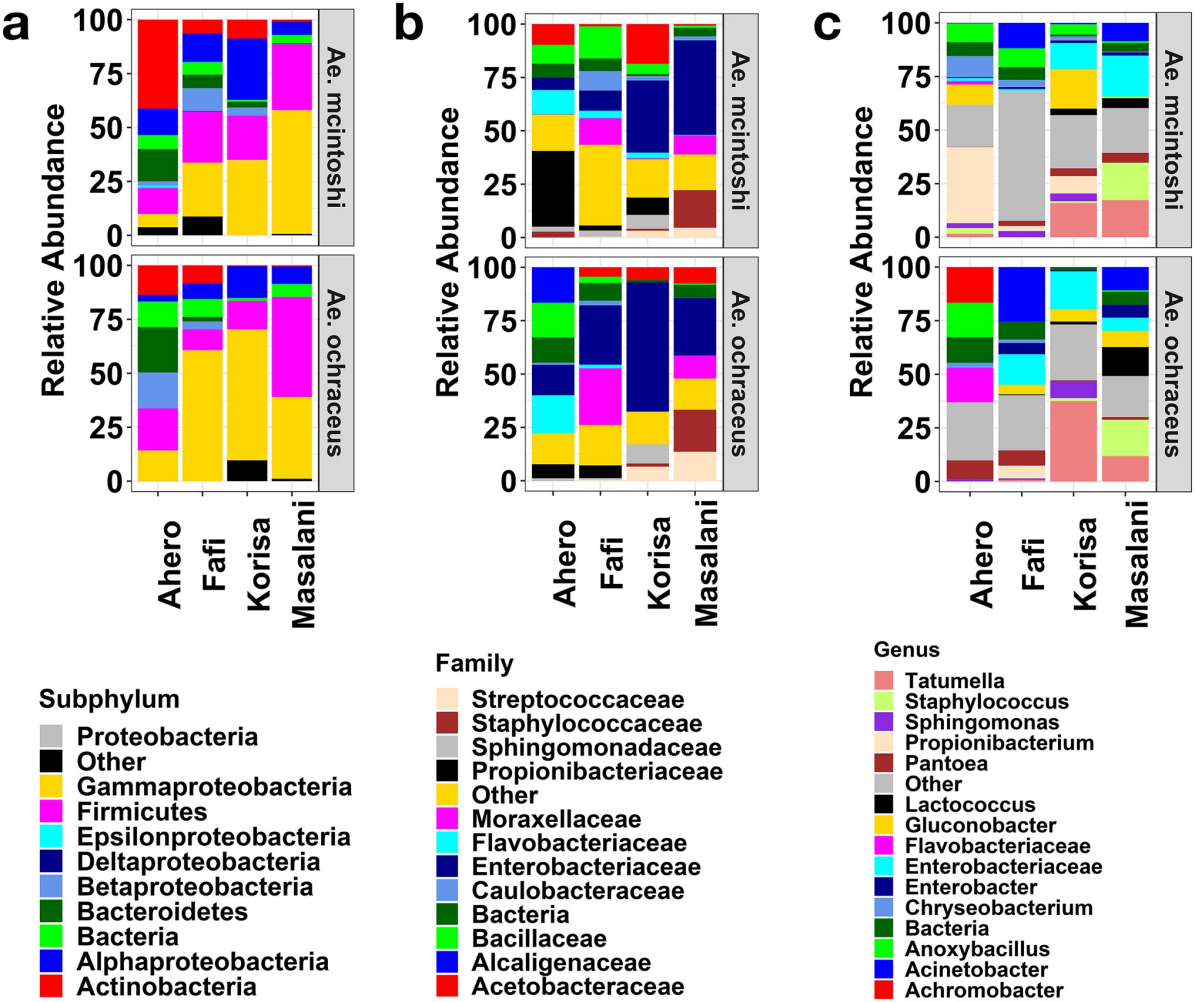
Mean relative abundances of bacterial OTUs associated with *Aedes mcintoshi* (upper panel) and *Aedes ochraceus* (lower panel) from the different study sites at the A) subphylum, B) Family and C) genus levels. Families and genera accounting for less than 2% of the total sequences were pooled as “other”.

A total of 10 bacterial phyla were detected in the two mosquito species (Fig 5a). The five most abundant phyla were Proteobacteria (53.5%), Firmicutes (22.0%), Actinobacteria (10.0%), Bacteroidetes (5.9%), and unclassified bacteria (5.6%) which collectively accounted for 97% of the total microbiota. Other bacterial phyla included Chloroflexi, Cyanobacteria, Fusobacteria, Planctomycetes and Tenericutes. Among the proteobacteria, Gammaproteobacteria (37.1%) and Alphaproteobacteria (11.7%) were the most dominant groups followed by Betaproteobacteria (4.6%). Gammaproteobacteria and Firmicutes were abundant in both mosquito species and their relative abundance varied markedly by study site (S1 Table). Actinobacteria occurred in both mosquito species but was more abundant in mosquito samples from Ahero especially *Ae*. *micintoshi*. The most abundant OTUs were from the families *Enterobacteriaceae* (27.9%), *Moraxellaceae* (7.5%), *Propionibacteriaceae* (7.3%), *Bacillaceae* (6.2%), *Acetobacteraceae* (6.0%), unclassified bacteria (5.6%), *Staphylococcaceae* (5.3%), *Flavobacteriaceae* (4.6%), *Streptococcaceae* (3.6%) and *Sphingomonadaceae* (2.8%) (Fig 5b). The relative abundance of the most common bacterial families varied markedly by mosquito species and study site (S2 Table). For example, *Propionibacteriaceae* were more abundant in *Ae*. *mcintoshi* from Ahero (35.5%) while *Enterobacteriaceae* were more abundant in *Ae*. *mcintoshi* from Korisa (33.8%) and Masalani (44.4%) and *Ae*. *ochraceus* from Fafi (27.8%), Korisa (60.6%) and Masalani (27%). *Flavobacteriaceae* were more abundant in mosquito samples from Ahero (17.9%). A summary of the comparisons in relative abundance of *Ae*. *mcintoshi* and *Ae*. *ochraceus* OTUs across the study sites are presented in S1 Table.

Similar variations in relative abundance of bacterial communities by species and study site were observed at genus level (Fig 5c). For example, *Propionibacterium* spp. and *Achromobacter* spp., respectively, were more abundant in *Ae*. *mcintoshi* (6.6%) and *Ae*. *ochraceus* from Ahero (16.6%) compared to other sites. Unclassified genera from family *Flavobacteriaceae* was more abundant in *Ae*. *ochraceus* from Ahero (16.2%) while *Lactococcus* spp. was more abundant in *Ae*. *ochraceus* from Masalani (13.6%). *Staphylococcus* spp. was more abundant in both mosquito species from Masalani (17–17.4%).

### Community structure

**Fig 6:**
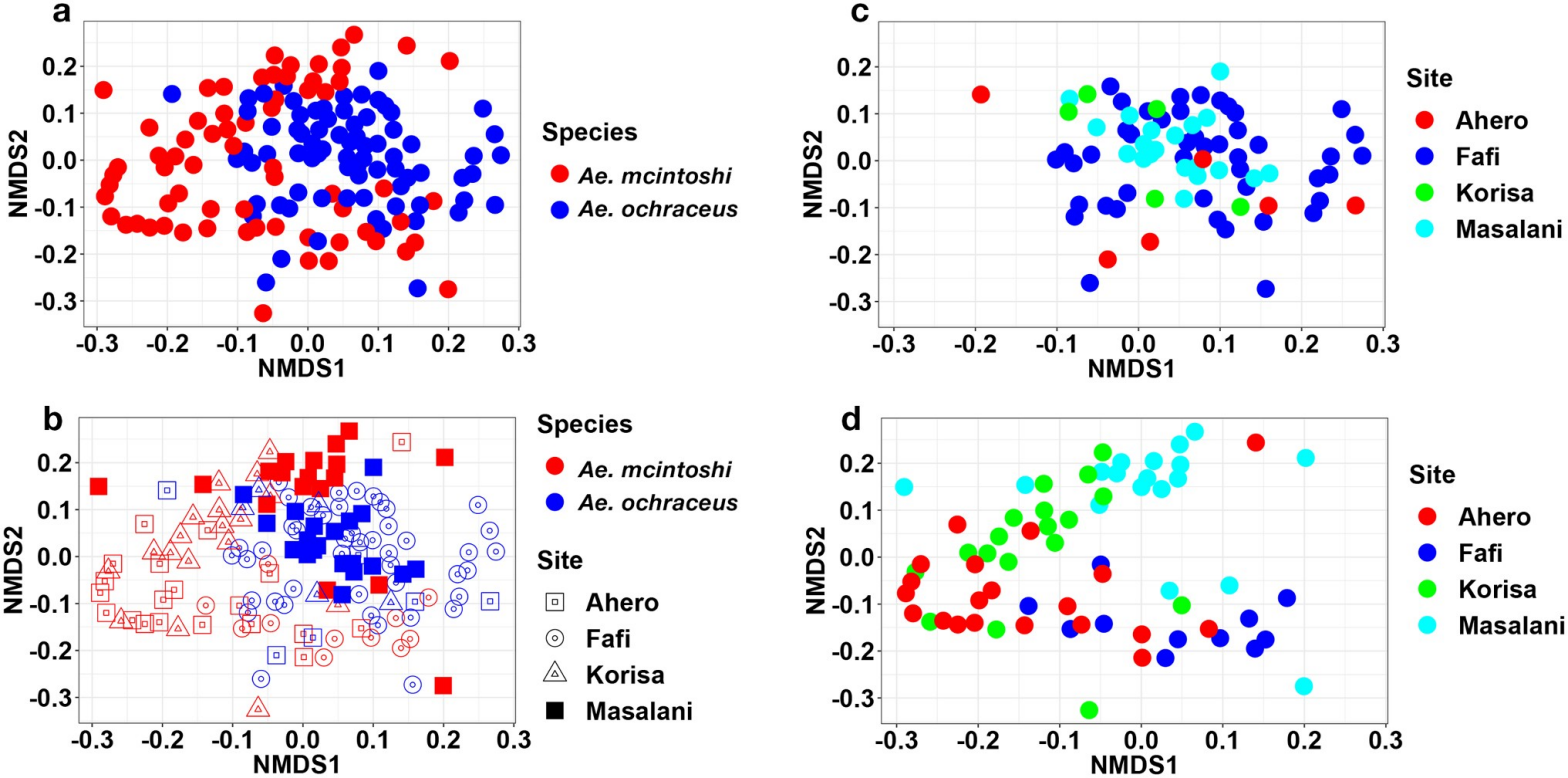
Nonmetric Multidimensional Scaling (NMDS) ordination displaying the microbial communities of *Aedes mcintoshi* and *Aedes ochraceus*. (a) both species from all sites pooled, (b) both species by study site, (c) *Ae*. *ochraceus* by study site and (d) *Ae*. *mcintoshi* by study site.

NMDS plots based on Bray-Curtis dissimilarities revealed a clear grouping of the samples by mosquito species with some degree of overlap (Fig 6a). These results were confirmed by ANOSIM test (R = 0.19, *P* = 0.0001). Additional analysis revealed a significant effect of study site on microbiota of the two mosquito species (Fig 6b, 6c and 6d). A follow up ANOSIM test showed that 20 out of the 28 pairwise comparisons were significantly different after Bonferroni correction for multiple comparisons (R = 0.37, *P* <0.0001). One pairwise comparison (OC-MA vs MC-FA) had an R value >0.75 indicating substantial differences in microbial community structure. Nineteen pairwise comparisons had R values ranging from 0.27–0.68 indicating varying degree of overlap but generally different community structure (Table 2). The remaining 8 pairwise comparisons had R values ≤0.24 indicating little separation.

### Indicator species analysis

Indicator species analysis revealed 7 bacterial genera that were strongly associated with either *Ae*. *mcintoshi* or *Ae*. *ochraceus* (Table 3). *Propionibacterium*, *Anoxybacillus*, *Chryseobacterium*, and *Roseomonas* were significantly associated with *Ae*. *mcintoshi* while *Enterobacter*, *Acinetobacter*, and unclassified bacteria were significantly associated with *Ae*. *ochraceus*. However, only the unclassified bacteria had the cutoff indicator value ≥60%.

**Table 3:** Results of indicator species analysis showing the microbial genera that were significantly associated with the two vectors of RVF virus.

Genus	Species	Indicator value	*P* value
*Propionibacterium*	*Ae*. *mcintoshi*	0.41	0.001
*Anoxybacillus*	*Ae*. *mcintoshi*	0.19	0.019
*Chryseobacterium*	*Ae*. *mcintoshi*	0.13	0.043
*Roseomonas*	*Ae*. *mcintoshi*	0.10	0.004
Unclassified bacteria	*Ae*. *ochraceus*	0.62	0.039
*Enterobacter*	*Ae*. *ochraceus*	0.51	0.003
*Acinetobacter*	*Ae*. *ochraceus*	0.46	0.001

## Discussion

We used amplicon-based MiSeq sequencing to characterize the bacterial communities of *Ae*. *mcintoshi* and *Ae*. *ochraceus*, the primary vectors of RVFV in Kenya. Our findings reveal that the two mosquito species harbor distinct microbial communities and that *Ae*. *ochraceus* has a more diverse microbial community compared to *Ae*. *mcintoshi*. The microbial communities of the two mosquito species were also strongly influenced by the site of collection. Proteobacteria (53.5%), Firmicutes (22.0%) and Actinobacteria (10.0%) were the most abundant phyla which is consistent with previous findings on microbiota of field-collected mosquitoes [17–20].

Variation in microbial composition between the two mosquito species is intriguing given that they were collected from the same study sites and are known to utilize similar aquatic habitats commonly referred to as dambos for oviposition and larval development [44]. They also exploit livestock hosts as the primary source of blood meals [45,46]. The findings may relate to differences in their biology and exploitation of resources as adults. Both species may have different physiologies that dictates which microbial species can colonize and thrive in their bodies. In addition, blood feeding and sugar feeding are common in these mosquito species [47], and both traits are known to influence the composition and diversity of microbial communities in mosquitoes [48]. Delineating the microbiota contribution of different blood meal and nectar feeding sources, would require additional research.

For each species, OTU richness varied by the site of mosquito collection suggesting an effect of local environment on microbial richness. Our findings are consistent with previous findings that the local environment is a key determinant of microbial communities in wild-caught mosquitoes [17,29,49]. This effect may partly be mediated by microclimatic differences among the sites such as temperature and humidity. These variables have been found to influence the abundance of microbial communities such as *Wolbachia* in *Culex* mosquitoes [50]. Also, plants serve as a food resource for adult mosquitoes and variation in the composition and diversity of plant communities between the study sites may influence bacterial composition, richness, and diversity in mosquitoes [49]. Contamination of aquatic habitats with pollutants such as pesticides may also alter the microbial communities in the aquatic habitats which in turn may influence the microbial communities that mosquitoes acquire from the larval environment [30]. However, we neither quantified the chemical contaminants that the mosquitoes were exposed to nor the plant communities that were present in our study sites. Future studies should include these aspects.

There is evidence that the two mosquito species differ in vector competence for RVFV as well as in their contribution to RVFV transmission in nature based on infection rates during the RVF outbreak of 2006/07 [24]. OTUs belonging to the bacterial genera *Propionibacterium*, *Anoxybacillus*, *Chryseobacterium*, *Roseomonas* were mostly associated with *Ae*. *mcintoshi*, while the genera *Enterobacter*, *Acinetobacter* and unclassified bacteria were mostly associated with *Ae*. *ochraceus* (Table 3). Bacterial species in some of these genera have been known to influence vector susceptibility to pathogens. For instance, *Enterobacter* spp. isolated from *Ae*. *albopictus* was shown to directly inhibit La Crosse virus in vitro assays [51]. Similarly, *Acinetobacter* spp. inhibited *P*. *falciparum* infection in mosquitoes [52]. Removal of midgut bacteria including *Chryseobacterium* via antibiotic treatment increased the susceptibility of *Anopheles gambiae* to *Plasmodium* infection [53,54]. Conversely, *Plasmodium* infection success in *An*. *gambiae* was associated with higher abundance of Enterobacteriaceae [17]. While bacteria in the genera *Propionibacterium*, *Anoxybacillus and Roseomonas* have been previously reported in other mosquitoes [20,55,56], little is known about their effects on pathogen transmission. Whether differences in the contribution of the two mosquito species to RVFV transmission in nature are related to their microbiota profiles remains unclear. Thus, assessing the impact of the identified bacteria on vector competence for RVFV should be the focus of future studies.

Some of the bacterial genera that were differentially abundant among study sites or between mosquito species included *Tatumella*, *Gluconobacter*, *Acinetobacter*, *Anoxybacillus*, *Lactococcus*, *Achromobacter*, *Staphylococcus*, *Proprionibacteria* among others. *Acinetobacter*, an acetic acid bacterium largely associated with floral nectar is among the core microbiota of many mosquito species and is likely acquired through sugar-feeding or contact with flowering plants [57,58]. *Gluconobacter* and *Roseomonas* are also acetic acid bacteria that are adapted to various sugar-rich and ethanol-rich environments [59] and are commonly found in insects that depend on sugar-based diets including mosquitoes [60]. *Achromobacter* and *Anoxybacillus* are commonly found in soil and water and adult mosquitoes may acquire them through contact with soil and water or through transstadial transmission [61]. *Tatumella* spp. has previously been described in other adult mosquito species [20]. Members of this genus along with those of genus *Lactobacillus* are involved in fermentation [62]. *Propionibacterium* include common bacteria of human skin and other animals and may be acquired when the mosquito lands on a blood-meal host.

In summary, we provide the first comprehensive report of the microbial communities of field-caught populations of the primary vectors of RVFV in Kenya. We show that the two mosquito species have distinct microbial communities whose diversity and richness is heavily influenced by the site of collection. Because vector susceptibility to pathogens may be influenced by certain bacterial species, further studies are warranted to investigate the functional role of identified microbiota on the biology of the mosquito species including influence on susceptibility to RVFV. These studies may propel identification of bacterial taxa that may be harnessed for symbiotic control of RVF virus.

## Supporting information

S1 Table Comparisons in relative abundance of *Aedes mcintoshi* and *Aedes ochraceus* OTUs across study sites. Multiple comparisons performed using chi square goodness-of-fit test with pairwise comparisons using adjusted *P* after *false discovery rate* (*fdr*) correction at α = 0.05.
(DOCX)

S2 Table Comparisons in relative abundance of OTUs between *Aedes mcintoshi* and *Aedes ochraceus* by study site. Comparisons performed using chi square goodness-of-fit test at α = 0.05.
(DOCX)
