# Supplementary material for: Host species and site of collection shape the microbiota of Rift Valley fever vectors in Kenya
Source: PLoS Negl Trop Dis. 2019 Jun 7;13(6):e0007361. doi: 10.1371/journal.pntd.0007361 (PMC6584011; doi:10.1371/journal.pntd.0007361)
Supplement: S1 Table — Multiple comparisons performed using chi square goodness-of-fit test with pairwise comparisons using adjusted P after false discovery rate (fdr) correction at α = 0.05. (DOCX) [file pntd.0007361.s001.docx]

|  |  | *Aedes mcintoshi* | | | | | | *Aedes ochraceus* | |  |  |  |  |
| --- | --- | --- | --- | --- | --- | --- | --- | --- | --- | --- | --- | --- | --- |
|  | **OTUs** | Ahero | Fafi | Korisa | Masalani | χ2 | P-value | Ahero | Fafi | Korisa | Masalani | χ2 | P-value |
| Subphylum | Gammaproteobacteria | 6.13c | 24.98b | 34.88b | 57.37a | 44.26 | <0.0001 | 14.25c | 60.69a | 60.588a | 37.9b | 34.01 | <0.0001 |
|  | Firmicutes | 12.12bc | 23.74ab | 20.58ab | 30.64a | 8.13 | 0.04 | 19.47b | 9.67b | 13.30b | 46.36a | 37.27 | <0.0001 |
|  | Alphaproteobacteria | 12.24b | 13.12b | 28.50a | 6.21b | 18.02 | 0.00 | 3.09b | 7.29ab | 14.78a | 8.19ab | 8.42 | 0.04 |
|  | Actinobacteria | 41.27a | 6.48b | 8.68b | 0.89c | 69.78 | <0.0001 | 13.66a | 8.31a | 0.30b | 0.44b | 22.37 | <0.0001 |
|  | Bacteroidetes | 14.99a | 6.24ab | 2.66bc | 0.29c | 20.62 | 0.0001 | 21.00a | 2.14b | 0.001b | 0.001b | 53.88 | <0.0001 |
|  | Bacteria | 6.49a | 5.93a | 0.89a | 3.89a | 4.48 | 0.21 | 11.90a | 8.28a | 1.31b | 6.02ab | 8.57 | 0.04 |
|  | Betaproteobacteria | 1.96b | 10.61a | 3.71ab | 0.001b | 15.7 | 0.001 | 16.62a | 3.63b | 0.001b | 0.004b | 36.9 | <0.0001 |
|  | Epsilonproteobacteria | 1.02 | 0.00 | 0.00 | 0.00 | na | na | 0.00 | 0.00 | 0.00 | 0.00 | na | na |
|  | Deltaproteobacteria | 0.00 | 0.15 | 0.00 | 0.00 | na | na | 0.00 | 0.00 | 0.00 | 0.00 | na | na |
|  | Proteobacteria | 0.00 | 0.00 | 0.09 | 0.00 | na | na | 0.00 | 0.00 | 0.00 | 0.00 | na | na |
|  | Other | 3.80ab | 8.74a | 0.02b | 0.70b | 14.3 | 0.003 | 0.01b | 0.004b | 9.71a | 1.09b | 24.5 | <0.0001 |
| Family | Enterobacteriaceae | 5.84b | 9.48b | 33.83a | 44.36a | 44.93 | <0.0001 | 14.25b | 27.81b | 60.56a | 27.00b | 36.187 | <0.0001 |
|  | Moraxellaceae | 0.17b | 12.61a | 0.58b | 8.88a | 20.61 | 0.0001 | 0.001c | 26.64a | 0.02c | 10.83b | 50.74 | <0.0001 |
|  | Propionibacteriaceae | 35.51a | 2.37bc | 8.14b | 0.003c | 69.82 | <0.0001 | 6.65a | 6.02a | 0.001b | 0.001b | 12.73 | 0.005 |
|  | Bacillaceae | 8.81ab | 15.02a | 4.83bc | 1.08c | 14.34 | 0.002 | 16.19a | 2.93b | 0.0001b | 0.73b | 34.81 | <0.0001 |
|  | Acetobacteraceae | 9.73a | 1.11b | 18.61a | 0.77b | 28.39 | <0.0001 | 0.001b | 4.45ab | 5.76a | 7.57a | 7.03 | 0.07 |
|  | Bacteria | 6.49a | 5.93a | 0.89a | 3.89a | 4.48 | 0.21 | 11.90a | 8.28ab | 1.31b | 6.02ab | 8.57 | 0.036 |
|  | Staphylococcaceae | 2.76b | 0.001b | 0.97b | 17.58a | 38.3 | <0.0001 | 0.01b | 0.05b | 1.51b | 19.74a | 52.26 | <0.0001 |
|  | Flavobacteriaceae | 11.36a | 3.36ab | 2.56b | 0.29b | 15.89 | 0.001 | 17.89a | 1.67b | 0.0001b | 0.001b | 46.46 | <0.0001 |
|  | Streptococcaceae | 0.001a | 0.35a | 3.19a | 4.60a | 7.32 | 0.06 | 0.001c | 0.54c | 6.59b | 13.58a | 23.35 | <0.0001 |
|  | Sphingomonadaceae | 2.32a | 2.92a | 6.42a | 0.001a | 7.25 | 0.06 | 1.10b | 0.59b | 9.02a | 0.02b | 20.18 | 0.0002 |
|  | Alcaligenaceae | 0.00 | 0.00 | 0.00 | 0.00 | na | na | 16.62a | 0.00001b | 0.00001b | 0.00001b | 49.86 | <0.0001 |
|  | Caulobacteraceae | 0.002b | 9.10a | 2.08ab | 1.86ab | 14.74 | 0.002 | 0.98a | 2.15a | 0.0001a | 0.04a | 3.88 | 0.28 |
|  | Other | 17.00b | 37.76a | 17.91b | 16.69b | 14.23 | 0.003 | 14.42a | 18.86a | 15.23a | 14.47a | 0.85 | 0.84 |
| Genus | Tatumella | 1.57b | 0.001b | 15.88a | 17.34a | 29.06 | <0.0001 | 0.001c | 0.82c | 37.42a | 11.81b | 73.06 | <0.0001 |
|  | Enterobacteriaceae | 1.52b | 0.77b | 12.17a | 19.17a | 28.04 | <0.0001 | 0.45c | 14.25a | 17.70a | 6.20ab | 18.91 | 0.00 |
|  | Acinetobacter | 0.17b | 11.80a | 0.58bb | 8.53a | 19.22 | 0.0002 | 0.001c | 25.48a | 0.02c | 10.82b | 48.07 | <0.0001 |
|  | Propionibacterium | 35.51a | 2.37bc | 8.14b | 0.003c | 69.83 | <0.0001 | 0.001b | 6.02a | 0.001b | 0.0005b | 18.05 | 0.00 |
|  | Gluconobacter | 9.38a | 0.002b | 18.38a | 0.76b | 31.28 | <0.0001 | 0.001b | 4.43ab | 5.69a | 7.49a | 6.94 | 0.07 |
|  | Bacteria | 6.49a | 5.93a | 0.89a | 3.89a | 4.48 | 0.21 | 11.90a | 8.28ab | 1.31b | 6.02ab | 8.57 | 0.04 |
|  | Anoxybacillus | 8.80a | 8.85a | 4.82ab | 0.98b | 7.25 | 0.06 | 16.14a | 0.001b | 0.001b | 0.73b | 45.01 | <0.0001 |
|  | Staphylococcus | 2.76b | 0.0001b | 0.97b | 17.43a | 37.89 | <0.0001 | 0.01b | 0.001b | 1.51b | 16.96a | 44.27 | <0.0001 |
|  | Pantoea | 0.10a | 2.41a | 3.58a | 4.64a | 4.24 | 0.24 | 8.77a | 7.12ab | 0.40c | 1.22b | 12.02 | 0.01 |
|  | Lactococcus | 0.0001a | 0.0001a | 3.05a | 4.60a | 8.28 | 0.05 | 0.001b | 0.43b | 1.39b | 13.58a | 33.05 | <0.0001 |
|  | Chryseobacterium | 9.76a | 3.31ab | 1.86b | 0.29b | 13.63 | 0.003 | 1.65a | 1.64a | 0.00a | 0.00a | 3.28 | 0.35 |
|  | Sphingomonas | 2.25a | 2.88a | 3.59a | 0.0001a | 3.32 | 0.35 | 1.09b | 0.56b | 7.86a | 0.02b | 17.03 | 0.00 |
|  | Flavobacteriaceae | 1.60a | 0.03a | 0.02a | 0.002a | 4.56 | 0.21 | 16.24a | 0.01b | 0.00b | 0.00b | 48.66 | <0.0001 |
|  | Achromobacter | 0.00 | 0.00 | 0.00 | 0.00 | na | na | 16.62a | 0.00b | 0.00b | 0.00b | 49.85 | <0.0001 |
|  | Enterobacter | 0.42a | 0.95a | 1.27a | 1.51a | 0.64 | 0.89 | 0.06a | 5.7a | 0.65a | 6.00a | 9.46 | 0.02 |
|  | Other | 19.69b | 60.70a | 24.81b | 20.86b | 36.49 | <0.0001 | 27.09a | 25.68a | 26.05a | 19.15a | 1.60 | 0.66 |
