# Supplementary material for: Host species and site of collection shape the microbiota of Rift Valley fever vectors in Kenya
Source: PLoS Negl Trop Dis. 2019 Jun 7;13(6):e0007361. doi: 10.1371/journal.pntd.0007361 (PMC6584011; doi:10.1371/journal.pntd.0007361)
Supplement: S2 Table — Comparisons performed using chi square goodness-of-fit test at α = 0.05. (DOCX) [file pntd.0007361.s002.docx]

|  |  | Ahero | | | Fafi | | | Korisa | | | Masalani | | |
| --- | --- | --- | --- | --- | --- | --- | --- | --- | --- | --- | --- | --- | --- |
|  |  | *Ae. mcintoshi* | *Ae. ochraceus* | P-value | *Ae. mcintoshi* | *Ae. ochraceus* | P-value | *Ae. mcintoshi* | *Ae. ochraceus* | P-value | *Ae. mcintoshi* | *Ae. ochraceus* | P-value |
| Subphylum | Gammaproteobacteria | 6.13a | 14.25a | 0.07 | 24.98b | 60.69a | 0.00 | 34.88b | 60.59a | 0.01 | 57.37a | 37.9b | 0.046 |
|  | Firmicutes | 12.12a | 19.47a | 0.19 | 23.74a | 9.67b | 0.01 | 20.58a | 13.30a | 0.21 | 30.64a | 46.36a | 0.07 |
|  | Alphaproteobacteria | 12.24a | 3.09b | 0.02 | 13.12a | 7.29a | 0.20 | 28.50a | 14.78b | 0.04 | 6.21a | 8.19a | 0.6 |
|  | Actinobacteria | 41.27a | 13.66b | 0.00 | 6.48a | 8.31a | 0.65 | 8.68a | 0.30b | 0.01 | 0.89a | 0.44a | 0.7 |
|  | Bacteroidetes | 15.00a | 21.00a | 0.32 | 6.24a | 2.14a | 0.16 | 2.66a | 0.00a | 0.10 | 0.29a | 0.001a | 0.59 |
|  | Bacteria | 6.49a | 11.90a | 0.21 | 5.93a | 8.28a | 0.53 | 0.89a | 1.31a | 0.77 | 3.89a | 6.02a | 0.5 |
|  | Betaproteobacteria | 1.96b | 16.62a | 0.00 | 10.61a | 3.63a | 0.06 | 3.71a | 0.00b | 0.05 | 0.00 | 0.00 | na |
|  | Epsilonproteobacteria | 1.02 | 0.00 | na | 0.00 | 0.00 | na | 0.00 | 0.00 | na | 0.00 | 0.00 | na |
|  | Deltaproteobacteria | 0.00 | 0.00 | na | 0.15 | 0.00 | na | 0.00 | 0.00 | na | 0.00 | 0.00 | na |
|  | Proteobacteria | 0.00 | 0.00 | na | 0.00 | 0.00 | na | 0.09 | 0.00 | na | 0.00 | 0.00 | na |
|  | Other | 3.79a | 0.01b | 0.05 | 8.74a | 0.003b | 0.00 | 0.02b | 9.71a | 0.00 | 0.70a | 1.09a | 0.77 |
| Family | Enterobacteriaceae | 5.84a | 14.25a | 0.06 | 9.48b | 27.81a | 0.00 | 33.83b | 60.56a | 0.01 | 44.36a | 27.00b | 0.04 |
|  | Moraxellaceae | 0.17 | 0.00 | 0.68 | 12.61b | 26.64a | 0.03 | 0.58a | 0.02a | 0.47 | 8.89a | 10.83a | 0.67 |
|  | Propionibacteriaceae | 35.51a | 6.65b | <0.0001 | 2.37a | 6.02a | 0.21 | 8.14a | 0.00b | 0.00 | 0.00 | 0.00 | na |
|  | Bacillaceae | 8.81a | 16.19b | 0.14 | 15.02a | 2.93b | 0.00 | 4.83a | 0.00b | 0.03 | 1.08a | 0.73a | 0.79 |
|  | Acetobacteraceae | 9.73a | 0.00b | 0.00 | 1.11a | 4.45a | 0.16 | 18.61a | 5.76b | 0.01 | 0.77b | 7.57a | 0.02 |
|  | Bacteria | 6.49a | 11.90a | 0.21 | 5.93a | 8.28a | 0.53 | 0.89a | 1.31a | 0.78 | 3.89a | 6.02a | 0.5 |
|  | Staphylococcaceae | 2.76a | 0.01a | 0.10 | 0.00 | 0.05 | 0.82 | 0.97a | 1.51a | 0.73 | 17.58a | 19.74a | 0.72 |
|  | Flavobacteriaceae | 11.36a | 17.89a | 0.23 | 3.36a | 1.67a | 0.45 | 2.56a | 0.00a | 0.11 | 0.29 | 0.00 |  |
|  | Streptococcaceae | 0.00 | 0.00 | na | 0.35a | 0.54a | 0.84 | 3.19a | 6.59a | 0.28 | 4.6b | 13.58a | 0.04 |
|  | Sphingomonadaceae | 2.32a | 1.10a | 0.51 | 2.92a | 0.59a | 0.21 | 6.42a | 9.02a | 0.51 | 0.00 | 0.02 | na |
|  | Alcaligenaceae | 0.00b | 16.62b | <0.0001 | 0.00 | 0.00 | na | 0.00 | 0.00 | na | 0.00 | 0.00 | na |
|  | Caulobacteraceae | 0.00 | 0.98 | 0.32 | 9.010a | 2.15b | 0.04 | 2.08a | 0.00a | 0.15 | 1.86a | 0.04a | 0.19 |
|  | Other | 17.00a | 14.42a | 0.65 | 37.76a | 18.86b | 0.01 | 17.91a | 15.23a | 0.64 | 16.69a | 14.47a | 0.69 |
| Genus | *Tatumella* | 1.57a | 0.00a | 0.21 | 0.00 | 0.82 | na | 15.88b | 37.42a | 0.00 | 17.34a | 11.81a | 0.31 |
|  | *Enterobacteriaceae* | 1.52a | 0.45a | 0.45 | 0.77 | 14.25 | 0.00 | 12.17a | 17.7a | 0.31 | 19.17a | 6.20b | 0.01 |
|  | *Acinetobacter* | 0.17 | 0.00 | na | 11.80 | 25.48 | 0.03 | 0.58 | 0.02 | na | 8.53a | 10.82a | 60 |
|  | *Propionibacterium* | 35.51a | 0.00b | <0.0001 | 2.37a | 6.02a | 0.21 | 8.14a | 0.00b | 0.00 | 0.00 | 0.00 | na |
|  | *Gluconobacter* | 9.38a | 0.00b | 0.00 | 0.002b | 4.43a | 0.04 | 18.38a | 5.69b | 0.01 | 0.76b | 7.49a | 0.01 |
|  | *Bacteria* | 6.49a | 11.90a | 0.21 | 5.93a | 8.28a | 0.53 | 0.89a | 1.31a | 0.78 | 3.89a | 6.02a | 0.5 |
|  | *Anoxybacillus* | 8.80a | 16.14a | 0.14 | 8.85a | 0.00b | 0.00 | 4.82a | 0.00b | 0.03 | 0.98a | 0.73a | 0.85 |
|  | *Staphylococcus* | 2.76a | 0.01a | 0.10 | 0.00 | 0.00 | na | 0.97a | 1.51a | 0.73 | 17.41a | 16.96a | 0.93 |
|  | *Pantoea* | 0.1b | 8.77a | 0.00 | 2.41a | 7.12a | 0.13 | 3.58a | 0.40a | 0.11 | 4.64a | 1.22a | 0.16 |
|  | *Lactococcus* | 0.00 | 0.00 | na | 0.00 | 0.43 | na | 3.05a | 1.39a | 0.43 | 4.60b | 13.58a | 0.04 |
|  | *Chryseobacterium* | 9.76a | 1.65b | 0.02 | 3.31a | 1.64a | 0.45 | 1.86a | 0.00a | 0.17 | 0.29 | 0.00 | na |
|  | *Sphingomonas* | 2.25a | 1.09a | 0.53 | 2.88a | 0.56a | 0.21 | 3.59a | 7.86a | 0.21 | 0.00 | 0.02 | na |
|  | *Flavobacteriaceae* | 1.60b | 16.24a | 0.00 | 0.03 | 0.01 | na | 0.02 | 0.00 | na | 0.00 | 0.00 | na |
|  | *Achromobacter* | 0.00b | 16.62a | <0.0001 | 0.00 | 0.00 | na | 0.00 | 0.00 | na | 0.00 | 0.00 | na |
|  | *Enterobacter* | 0.42 | 0.06 | na | 0.95a | 5.27a | 0.08 | 1.27a | 0.65a | 0.65 | 1.51a | 6.00a | 0.1 |
|  | Other | 19.69a | 27.09a | 0.28 | 60.70a | 25.68b | 0.00 | 24.81a | 26.05a | 0.86 | 20.86a | 19.15a | 0.79 |
